# The impact of attitudes as a mediator between sense of autonomy and help-seeking intentions for self-injury

**DOI:** 10.1186/s13034-015-0058-3

**Published:** 2015-07-08

**Authors:** Megan Pumpa, Graham Martin

**Affiliations:** Department of Psychiatry, The University of Queensland, K Floor, Mental Health Building, RBWH, Herston, 4006 Brisbane

**Keywords:** Self-injury, Self-harm, Help-seeking, Autonomy, Attitudes, Mediation analysis, Boot-strapping

## Abstract

**Background:**

Self-injury is a complex issue, further complicated by the fact that up to half of young people who self-injure do not receive help. Young people who do receive help for self-injury claim they prefer to access family and friends over more formal sources of help. This original research set out to examine the influence of negative attitudes to professional help and a sense of autonomy on help-seeking intentions.

**Methods:**

A cross-section of 220 university students and young adults from the community (Students = 131, other participants = 89; mean age = 24.64) completed anonymous online questionnaires measuring self-injurious behaviour and mental health related problems, attitudes toward seeking professional mental health help, autonomy, and intentions to seek help for self-injury. Two separate mediation models were tested using a bootstrapping approach to investigate intentions to seek help – one on mental health problems, and one specifically on self-injury.

**Results:**

More positive attitudes to help-seeking were significantly associated with greater intentions to seek help, while higher perceived autonomy was associated with lower intentions to seek help. Attitudes fully mediated the negative relationship between autonomy and willingness to seek help for self-injury. The model also maintained partial mediation for willingness to seek help for other mental health problems, beyond self-injury. Current self-injurers expressed significantly more negative attitudes toward help-seeking compared to past self-injurers and those with no history of self-injury. Similarly, current self-injurers reported being less likely to seek help from anyone compared to both other groups.

**Conclusions:**

This study appears to be the first to set out to compare self-injurers’ attitudes to help-seeking directly with those of non-self-injurers, and the first to show that attitudes mediate the relationship between autonomy and help-seeking. The findings provide evidence that will assist development of interventions targeting negative attitudes toward seeking professional help, in order to increase help-seeking among self-injurers who would otherwise not receive treatment.

## Introduction

In a recent systematic review, Rowe *et al.* [1] concluded that up to half the adolescents who self-injure do not seek help; but if they do, they are more likely to approach friends and family, especially if they are female. Only six of the nineteen studies included explored barriers to seeking help. Not knowing where to get help or what to expect from the help they might receive [2], and having practical access problems, for instance in rural areas [3], were major factors. Intrapsychic issues were common, including fears that professionals or parents might not be able to help [4], fear of loss of confidentiality or of being stigmatised [5], with this accentuated by poor mental health or suicidal ideation [6]. In addition, young people tended to minimise the seriousness of their self-injury, and believed they could or should manage alone [7]. Only two studies reported possible facilitators for help-seeking [2, 4]. Large scale cross-national research confirms that almost half of young self-injurers do not receive help from anyone [8], and this reluctance to seek help for self-injury may be more prominent in younger age groups [9], with young people often relying on the ability of others to recognise their troubles and respond accordingly, rather than actively seeking help [10].

Attitudinal barriers deterring young people from seeking help appear to be more important than practical barriers [11]. In fact, perceived social stigma around seeking help for mental health problems has been conceptualised as one of the greatest barriers to getting psychological treatment [12]. Conversely, lower perceived stigma has been shown to be associated with more positive attitudes toward seeking psychological help [13]. Symptom severity may not be associated with help-seeking, suggesting social stigma and negative attitudes together may prevent people from seeking psychological help, regardless of how severe the problem [13, 14]. Conversely, positive attitudes toward seeking professional mental health help predict having actually sought help from a General Practitioner [15]. Long *et al.* [16] suggest self-injury is hidden due to awareness of the social stigma specifically surrounding purposely damaging one’s body, and further, that awareness of stigma and resentment surrounding self-injury fosters a cycle of guilt and shame within the self-injurer, further fuelling the behaviour.

The self-injury literature often refers to shame, guilt, negative attitudes and stigma, but it has been unclear exactly how, and to what extent, attitudes influence help-seeking behaviours of self-injurers, despite being repeatedly shown to be a powerful barrier to seeking professional help for other mental health problems [12–14, 17, 18]. In the current study, we hypothesized that more positive attitudes would be associated with greater intentions to seek help for self-injury (Hypothesis 1). To our knowledge, the attitudes of self-injurers have never been compared directly to those of non-self-injurers. We further hypothesized that current self-injurers would report more negative attitudes toward seeking help for self-injury and mental health problems than either those with no history of self-injury, or those who had ceased self-injury for more than 12 months (‘past self-injurers’) (Hypothesis 2).

There is little research examining the effect autonomy has on willingness to seek help for mental health problems, though it has been suggested normal developmental needs of young people to become autonomous may interfere with help-seeking [18]. Greater need for autonomy is associated with lower intentions to seek help for a variety of common mental disorders, including anxiety and affective disorders [19]. Conversely lower perceived need for autonomy is associated with stronger intentions to seek professional mental help at some point in the future [20]. Need for autonomy appears to be a stronger barrier to help-seeking than help-seeking fears, for a range of other mental disorders including suicidal ideation, emotional problems, affective, and anxiety disorders [19, 20].

Given the existing research, the current study hypothesized autonomy would directly influence intentions to seek help for self-injury, but would also have an indirect effect on help-seeking via attitudes toward seeking help (Hypothesis 3). This research is the first to examine the impact of autonomy on help-seeking for self-injury.

Based on research showing self-reliance and the belief that self-injury can be managed without help are barriers to seeking help for self-injury [5, 9], we predicted that autonomy and attitudes toward seeking help would be associated, such that those with a greater autonomy need would also report more negative attitudes toward seeking help for mental health problems.

Finally we predicted attitudes would mediate the effect of the need for autonomy on help-seeking intentions, such that attitudes toward seeking mental health help would explain the existing relationship between autonomy and help-seeking intentions. Overall, this research aimed to highlight how key barriers prevent self-injurers from seeking help, to inform development of interventions to increase the proportion of self-injurers who receive help.

## Method

### Procedure

Ethics approval was gained from the University of Queensland BSSER Committee prior to data collection. A project summary was uploaded to the Sona system under the title: ‘Seeking help for mental health problems’. Participation in the study was voluntary and anonymous to ensure participants felt confident responding honestly to sensitive material. On completion student participants were given a debrief sheet and the opportunity to ask questions about the research and survey materials and discuss their reactions to the survey. Participants recruited via the Internet were automatically directed to an online link that provided an information sheet and debrief page. The online survey took approximately 10–15 min to complete.

### Participants

After discarding 16 partially completed surveys, a total of 220 participants completed the study. Forty participants were male (18.2 %), 179 female (81.4 %), with one claiming to be neither. More than half the participants (*n* = 131, 59.5 %) were current University of Queensland (UQ) students recruited through the UQ Sona system. Students participating in introduction to psychology courses are able to participate in research in exchange for course credit. Other participants (*n* = 89, 40.5 %) gained access to the survey through flyers posted on information boards throughout the UQ St. Lucia campus, or through online distribution of the study information on social media pages Facebook and Twitter. Participation was voluntary and anonymous for all participants. Overall, participants ranged from 15–64 years (mean 24.64 years, *SD* = 9.78). The majority of participants listed English as their primary language (90 %). Other languages spoken at home included Chinese, Hungarian, Mandarin, and Vietnamese.

### Measures

#### Demographics

Participants recorded age in years, sex, country of birth, primary language spoken, and if they were a current UQ student.

#### Autonomy Need

Autonomy need was measured using the Need for Autonomy (NA) subscale of the brief version of the **Barriers to Adolescents Seeking Help scale** (BASH-B) [20]. The subscale consists of two items measured on a 5-point Likert scale, ‘I think I should work out my own problems’ and ‘If I had a problem, I would solve it myself’. Responses ranged from ‘Strongly Disagree’ to ‘Strongly Agree’. The two items were combined to form a score of perceived need for autonomy, higher scores indicating greater perceived autonomy. In the current study the two items yielded an internal reliability coefficient of .73, within the range .70-.78 found by other studies [19, 20].

#### Self Injury

Participants Brief Self-Injury Questionnaire [21]. They first read a working definition of self-injury, before being asked ‘Have you ever hurt yourself on purpose? (If only once, please circle ‘yes’)’ Participants answering ‘no’ automatically bypassed the detailed self-injury section, and completed the remaining general scales. Those answering ‘yes’ were asked ‘Have you self-injured in the last 12 months?’ Those answering ‘yes’ were subsequently classified ‘current self-injurers’; those answering ‘no’ were classified as ‘previous self-injurers’. These groupings allow comparison of ‘current’, ‘previous’, and ‘no history of self-injury’ groups on all focal variables.

Items that followed assessed frequency of self-injury, forms of self-injury, functions of self-injury, whether anyone else was aware of their self-injury, whether help was received for self-injury, and if ‘yes’, the source of the help. Finally, self-injurers were asked whether their self-injury ‘was ever to suicide?’ and if ‘yes’, how many times they had attempted suicide.

The **Attitudes Toward Seeking Professional Help Scale** (ATSPHS) [22] contains 29 items, with responses on a 5-point Likert scale from ‘Strongly Disagree’ to ‘Strongly Agree’, and a neutral ‘neither agree nor disagree’ midpoint. Eighteen items are framed negatively (eg. ‘Having been a psychiatric patient is a blot on a person’s life’) with 11 items framed positively (eg. ‘If I thought I needed psychiatric help, I would get it no matter who knew about it’). Negative items were reverse scored, and the 29 items summed (range 58–133, mean 98.17), higher scores representing more positive attitudes. Our internal reliability (.90) was consistent with the original study (.86) [22].

The **General Help-Seeking Questionnaire** (GHSQ) [23] measures intention to seek help from a range of sources (from ‘intimate partner’ and ‘family member’, through ‘phone helpline’, to ‘doctor/GP’) for a range of different problems. It has predictive and construct validity, and good test-retest reliability for each of two core items, suicidal ideation, *r* = .88 and emotional problems, *r* = .86 [23]. Responses are on a 7-point Likert scale from 1 (Extremely Unlikely) to 7 (Extremely Likely), higher scores indicating a greater likelihood of seeking help from that source.

We added a third core item to assess help-seeking in relation to self-injury, using the original matrix format. Responses were categorised as ‘formal’ or ‘informal’ sources of help. Help-seeking from ‘informal’ and ‘formal’ sources for emotional problems, suicidal ideation, and self-injury combined yielded an internal reliability coefficient of .91 and .87 respectively.

To form a general score of intention to seek help, scores on the three ‘I would not seek help from anyone’ items were combined, higher scores indicating lower intentions to seek help. Our internal reliability coefficient, for the 3 combined items, was .88.

The **General Health Questionnaire** (GHQ-28) [24] contains a total of 28 items, measured on 4-point Likert scales. Participants were asked to answer items in reference to present and recent complaints. Four subscales (somatic symptoms, anxiety and insomnia, social dysfunction, and depression), with seven items each, yielded internal reliability coefficients, respectively, of .85, .90, .84, and .92. Individual subscales were combined to form a Total Score.

## Results

Of 220 participants, 7 failed to answer at least one question on the survey. No single variable was missing more than 5 % of data. Little’s (MCAR) test found missing values were randomly distributed across the dataset, *χ*^2^ (33, *N* = 220) = 42.77, *p* = .119. Missing values were therefore substituted using Expectation Maximisation (EM).

### Self-Injury

Overall, 48.6 % of participants (*n* = 107) reported a history of self-injury. Of males, 35.0 % had self-injured at least once in their life, compared to 51.4 % of females. There was no significant association between sex and prevalence of self-injury, *χ*^*2*^ (1, *n* = 219) = 2.89, *p* = .089, *phi* = −.13. Forty-eight participants (21.8 %) were ‘currently self-injuring’ and 58 participants were ‘previous self-injurers’ (no self-injury for at least 12 months).

Cutting was the most common method of self-injury (*n* = 69) followed by hitting self (*n* = 41) and wound picking (*n* = 32). Among self-injurers, 23.4 % had attempted suicide (*n* = 25), and 76.6 % reported nonsuicidal self-injury (*n* = 82). Independent samples *t*-test revealed self-injurers who had attempted suicide did not significantly differ from those denying suicide on variables of interest (GHQ, attitudes, help-seeking, and autonomy need) (see Table 1). All participants who had self-injured were included in subsequent analyses.

**Table 1:** Independent Samples T-tests Comparing Self-injurers who had and had not Attempted Suicide

Variables	Attempted suicide	Mean	*t*	df	*p* (2-tailed)
Attitudes	Yes	3.30	-0.23	105	.820
	No	3.34			
Autonomy	Yes	4.00	0.50	105	.619
	No	3.91			
Help-Seeking	Yes	3.80	1.10	32.16	.279
	No	3.25			
GHQ	Yes	2.39	1.08	105	.282
	No	2.25			

#### Receiving Help for Self-Injury

Of the sample of self-injurers (107), sixty-three reported someone was aware of their self-injury (59 %). Only forty-two of all self-injurers reported receiving help for self-injury (39.3 %). Of these, thirty-seven (88 %) received help from professional services including doctors or counsellors, twenty-one (50 %) reporting additional help from family or friends. Self-injurers who did (*M* = 2.30, *SD* = 0.65) and did not receive help (*M* = 2.27, *SD* = 0.54) for self-injury did not differ significantly on severity of symptomatology, *t*(105) = 0.28, *p* = .783 or sex, *χ*^*2*^ (1, *n* = 106) = 2.91, *p* = .509, *phi* = −.08.

#### Attitudes

Males (*M* = 3.16, *SD* = 0.51) reported significantly more negative attitudes toward seeking help for mental health problems than females (*M* = 3.43, *SD* = 0.57, *t*(217) = −2.80, *p* = .006, η^2^ = .03). Overall, males and females with more negative attitudes were more likely to report they would not seek help from anyone, r = −.46, p < .001. A one-way between-groups ANOVA with Bonferroni correction found attitudes toward seeking professional mental health help differed significantly by history of self injury, (*F*(2, 216) = 5.02, *p* = .007, η^2^ = .04). Multiple comparisons revealed current self-injurers (*M =* 3.17, *SD* = 0.65) reported significantly more negative attitudes toward seeking help for mental health problems than previous self-injurers (*M =* 3.47, *SD* = 0.55), (*t*(216) = −2.83, *p* = .015) and those with no history of self-injury (*M =* 3.44, *SD* = 0.51), (*t*(216) = −2.87, *p* = .014). An independent samples *t*-test comparing attitudes showed self-injurers receiving help for self-injury (*M* = 3.48, *SD* = 0.63) reported significantly more positive attitudes toward seeking help for mental health problems compared to those not receiving help (*M* = 3.23, *SD* = 0.58), (*t*(105) = 2.07, *p* = .041, η^2^ = .04).

#### Need for Autonomy

Autonomy did not vary by history of self injury, *F*(2, 216) = 0.80, *p* = .453. However, overall there was a negative association between need for autonomy and attitudes toward seeking help for mental health problems, with higher autonomy associated with more negative attitudes (*r* = −.28, *p* < .001). In addition, those higher in autonomy were more likely to report they would not seek help from anyone, (*r* = .28, *p* < .001).

#### Help-Seeking

A one-way between-groups ANOVA with Bonferroni corrections found intentions to seeking help for mental health problems differed significantly by history of self-injury (*F*(2, 216) = 12.11, *p* < .001, η^2^ = .10). Post Hoc comparisons revealed current self-injurers (*M =* 3.93, *SD* = 2.10) were significantly less likely to seek help for mental health problems, compared to previous self-injurers (*M =* 2.91, *SD* = 1.54, *t*(216) = 3.07, *p* = .007) and those with no history of self-injury (*M =* 2.49, *SD* = 1.57, *t*(216) = 4.92, *p* < .001). Self-injurers receiving help for self-injury (*M* = 3.07, *SD* = 2.21) reported significantly higher intentions to seek help for mental health problems compared to self-injurers not receiving help (*M* = 4.03, *SD* = 1.99, *t*(105) = −2.33, *p* = .022, η^2^ = .05). Participants for the sample as a whole were significantly more likely to seek help from informal sources (*M* = 51.25, *SD* = 16.55) than formal sources (*M* = 42.96, *SD* = 14.43, *t*(219) = 6.90, *p* < .001). Bivariate correlations suggested older participants were more likely to seek help (*r* = −.14, *p* = .046).

#### Mediation Analysis

Two separate mediation models were tested. The first examined attitudes as a mediator between autonomy and help-seeking for a broader range of mental health issues, including emotional problems and suicidal ideation. This model included the two ‘I would not seek help from anyone’ core items from the original GHSQ scale. The second model examined attitudes as a mediator between autonomy and help-seeking for our core question on self-injury. Each model was tested with and without controlling for covariates; there were no substantiative changes to findings.

The SPSS ‘mediate’ macro for bootstrapping mediation [25] was used to analyse the model to determine if attitudes mediated between autonomy and help-seeking for a range of mental health problems. This was chosen because it estimates both direct and indirect effects [26] and yields more power to find indirect effects than the causal steps approach developed by Baron & Kenny [27] [28]. ‘Perceived autonomy’ was entered as the predictor variable, ‘attitudes to seeking help for mental health problems’ as the proposed mediator, and ‘intentions to seek help’ as the dependent variable. For intercorrelations between predictor, mediator, outcome, and covariate variables please see Table 2.

**Table 2:** Means, Standard Deviations, and Inter-Correlations Between Variables in Analyses

Variables	*M*	*SD*	1	2	3	4	5	6	7
Age	24.62	9.80							
Sex	1.82	0.39	.02						
Attitudes	3.39	0.56	.28**	.19**					
Autonomy	3.93	0.76	-.04	-.11	-.28**				
Help-seeking^a^	2.92	1.77	-.14*	-.03	-.46**	.28**			
GHQ	2.02	0.58	-.08	.10	-.35**	.07	.42**		
Help for SI^b^	3.03	2.01	-.07	.02	-.41**	.20**	.88**	.39**	
GHSQ^c^	5.57	3.49	-.15*	-.08	-.45**	.32**	.93**	.36**	.80**

^a^ General measure of help-seeking. ^b^ Measure of help-seeking for self-injury. ^c^ Help-seeking for emotional problems and suicide ideation as measured by the GHSQ

* *p* < .05 ** *p* < .01

**Fig. 1:**
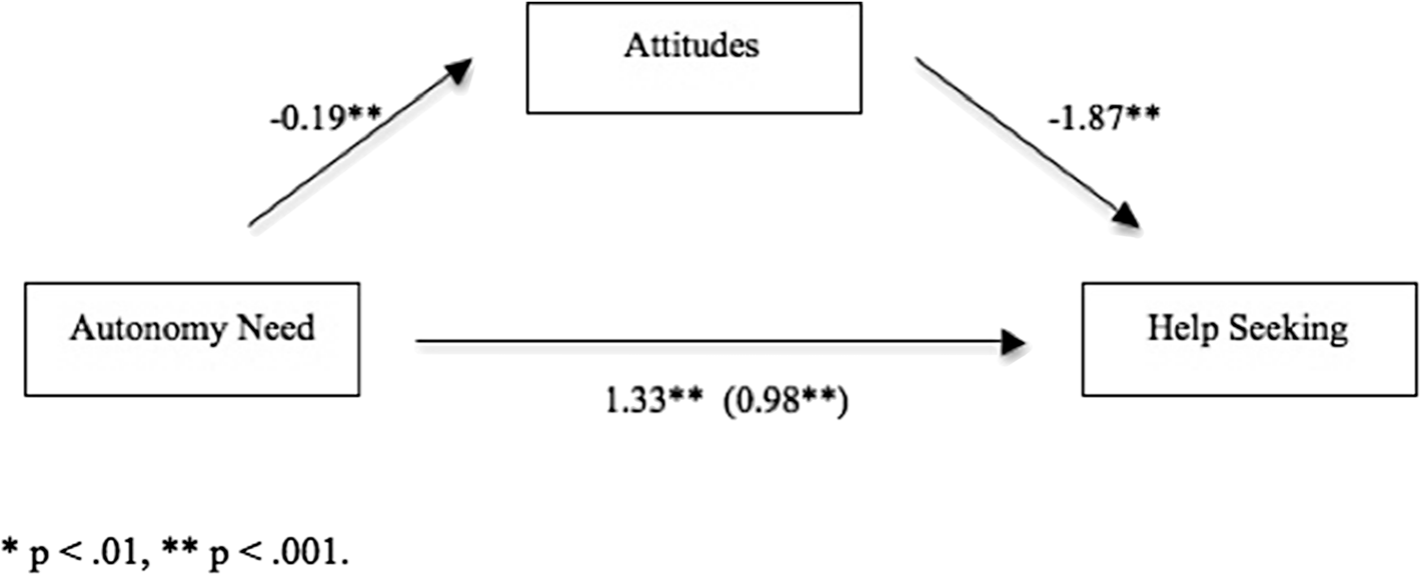
Bootstrapping results, with unstandardised coefficients, illustrating attitudes as a partial mediator of the autonomy and help-seeking relationship (Mental Health problems). * p < .01, ** p < .001

Age and symptomatology were controlled for as covariates to discount them as alternative explanations for findings [25]. Coefficients were estimated from 10,000 bootstrap samples, with replacement. The 95 % bias corrected confidence interval for the indirect effect did not include zero (lower bound = .1323, upper bound = .6208), indicating it was significantly different from 0 at *p* < .05. A test of homogeneity of regression was non-significant (*R*^*2*^ *=* .001, *F*(1, 213) = .17, *p* = .685), indicating autonomy and attitudes did not interact to affect help-seeking. The total (path c) and direct effects (path c^*I*^) of perceived autonomy on intentions to seek help were *b* = 1.33, *p* < .001 and *b* = 0.98, *p* > .001, respectively. Attitudes partially mediated the relationship between autonomy and help-seeking, the model accounting for 28 % of the variance in help-seeking (*Adj R*^*2*^ = .28 *F* (4, 214) = 21.69, *p* < .001). Participants reporting high levels of autonomy were more likely to have negative attitudes toward seeking help, and they also reported lower intentions to seek help. This relationship and the results of the bootstrapping analysis are represented visually in Fig. 1.

**Fig. 2:**
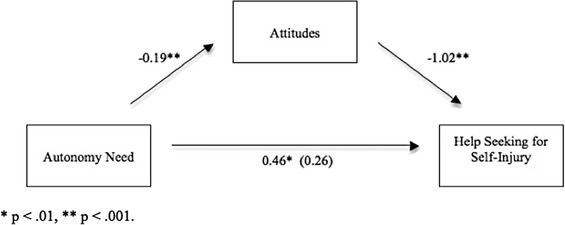
Bootstrapping results, illustrating attitudes as a mediator of the autonomy and help-seeking for self-injury relationship. * p < .01, ** p < .001

In the second mediation model symptomatology was again controlled for as a covariate. ‘Perceived autonomy’ was entered as the predictor variable, ‘attitudes’ as the mediator, and ‘intentions to seek help for self-injury’ as the dependent variable. Coefficients were estimated from 10,000 bootstrap samples, with replacement. The test of homogeneity of regression was non-significant (*R*^*2*^ *=* .001, *F*(1, 215) = .15, *p* = .704). The 95 % bias corrected confidence interval for the indirect effect did not include zero (lower bound = .0762, upper bound = .3465), indicating the indirect effect was significantly different from 0 at *p* < .05. This model accounted for 24 % of the variation in help-seeking for self-injury (*Adj R*^*2*^ = .24 *F* (3, 216) = 23.75, *p* < .001). The total (path c) and direct effects (path c^*I*^) of perceived autonomy on intention to seek help were *b* = 0.46, *p* = .006 and *b* = 0.26 (*p* = .113) respectively, suggesting attitudes fully mediated the relationship between autonomy and help-seeking for self-injury. Attitudes account for the negative relationship between high levels of autonomy and low intentions to seek help for self-injury. This relationship is represented visually in Fig. 2

## Discussion

This research investigated the role of attitudes toward seeking professional help, and autonomy, on help-seeking for self-injury. Key findings supported our original hypotheses that attitudes toward seeking help would mediate the relationship between autonomy and intentions to seek help for self-injury. In addition, differences were found between current and past self-injurers, compared with those with no history of self-injury. The findings highlight important differences between the groups that have implications for intervention and future research.

There were no significant differences in prevalence of self-injury between males and females in this study, which supports recent epidemiological research [29] and refutes older studies that may have limited questions to female methods such as cutting. The current study was not epidemiological, and deliberately set out to gain as many self-injurers as possible; no conclusions should be drawn as to community rates, or university and college rates, from the 48.6 % lifetime rate of self-injury for the sample.

Self-injurers who claimed they had also attempted suicide did not differ in terms of their attitudes, autonomy, intentions to seek help, or level of symptomatology as measured by the GHQ. This finding is surprising, as NSSI has recently been distinguished from self-injury *with* suicidal intent on a number of parameters, including depression measured by the GHQ [30]. As self-injurers who had also attempted suicide did not differ from participants engaging in NSSI alone, there was no significant reason to exclude them from our further analysis. While at the outset we focussed on the term ‘self-injury’, conclusions from the current study may also be applicable to those who ‘self-harm’ (a broader term which by definition includes attempts at suicide) [31].

Confirming previous research [19, 20], greater need for autonomy in our subjects was associated with being less willing to seek help for a range of mental health problems, and this finding extended to self-injury. This research is the first study to investigate the relationship between autonomy and attitudes toward seeking help in self-injurers. In line with hypotheses, a unique finding was that those reporting higher need for autonomy were also more likely to express more negative attitudes toward seeking professional mental help. For those needing to experience their actions as autonomous and self-initiated, getting help for a problem they believe they can control may threaten their sense of autonomy. The higher the sense of autonomy the more negative attitudes towards getting help may become.

Negative attitudes are a well-established barrier to seeking help for mental health problems [12, 15, 17]. In line with our hypotheses and based on this previous work, the current research found negative attitudes toward seeking help were associated with lower intentions to seek help for self-injury and other mental health problems. Again, our research is the first to seek to compare attitudes toward help-seeking of current self-injurers, previous self-injurers and those with no history of self-injury. In line with predictions, people currently self-injuring reported more negative attitudes toward seeking help for mental problems than past self-injurers and non-self-injurers. This is a unique finding of the current study, but supports existing literature [16] suggesting that awareness of stigma and feelings of guilt and shame experienced with self-injury may increase negative attitudes toward seeking help, and create a barrier. The causal direction of this cannot be determined without a longitudinal research design. Negative attitudes reduce with cessation of self-injury to the extent previous self-injurers are indistinguishable from non-self-injurers in terms of their attitudes toward seeking professional mental health help. This finding suggests that changing community and self-injurer attitudes to seeking help may be a fruitful direction in prevention and therapy.

Of participants with a history of self-injury, fewer than 40 % had received help for their self-injury at some point in the past, but those who had received help reported more positive attitudes toward seeking help than self-injurers who had not received help. Again, due to the cross-sectional nature of the study, we do not know if self-injurers received help because they started with more positive attitudes toward help-seeking, or whether more positive attitudes derive from having received help, and evaluating this experience as favourable.

The fact that over 60 % of self-injurers reported they had not received help concurs with other studies [8]. A unique finding of this study, however, was that current self-injurers were less likely to seek help for both self-injury and other mental health problems than previous self-injurers and non-self-injurers. This indicates those who are currently self-injuring and in need of help are the least likely to receive it. The finding further suggests that cessation of self-injury may be influenced by changed intentions to seek help.

Current self-injurers reported more mental health problems than previous self-injurers, who in turn reported more symptoms than non-self-injurers. However, symptom severity as such was not related to intention to seek help, nor to whether self-injurers had previously received help. This is consistent with findings that symptom severity and distress are not related to help-seeking [13].

There is general consensus within the literature that adolescents prefer to seek help for mental health problems from informal sources (family or friends) rather than professional services (hospitals and psychologists) [5, 9, 32]. While our study included a wider range of ages, it did confirm this; overall participants were more likely to seek help from informal sources than formal sources.

The main finding from this research was the novel mediation model examining attitudes as a mediator between autonomy and intentions to seek help. In line with predictions, attitudes fully mediated this relationship in the context of self-injury, suggesting autonomy has an indirect influence on intentions to seek help for self-injury. As self-injury is socially stigmatised, and often accompanied by shame and secrecy, it is perhaps not surprising that attitudes play such an important role. The need for autonomy may not be as malleable as attitudes; therefore interventions to change the influence of attitudes on help-seeking may be a more plausible and fruitful direction in therapy or prevention. Preliminary evidence does exist to suggest that attitudes can be changed, for instance by an education program in schools [33]. By implementing such strategies to improve attitudes toward seeking mental health and address existing negative beliefs about seeking mental health help, it may be possible to increase the proportion of self-injurers who seek help.

When the model was replicated for intentions to seek help for suicidal ideation and emotional problems more broadly, it remained significant for partial mediation. Whilst specifically relevant to self-injury, implications are that strategically influencing negative attitudes to improve help-seeking for mental health problems may have ramifications beyond self-injury.

Our research was not free of limitations. Our sample was self-selected, and may not represent the vast majority of community based self-injurers. As noted in epidemiological studies [29], the sex of participants is skewed, with more females than males participating. However, the high percentage of male self-injurers in the current study may not reflect gender ratios of self-injury in the community, and may have skewed our results, given males tend to report more negative attitudes toward help-seeking than females. Future research to corroborate our work may need to use broader recruitment methods to assess help-seeking for self-injury for the general population.

Due to the cross sectional nature of this research, it is not possible to infer causation between the measured variables and intentions to seek help. Differences between current and previous self-injurers do suggest changes occur concurrently with the cessation of self-injury. A longitudinal research design following self-injurers over time is needed to determine causal inferences between autonomy, attitudes, and help-seeking.

## Conclusions

The current study drew on qualitative studies of the role of attitudes in help-seeking for self-injury, but utilised standardised measures, adding to the limited literature on help-seeking for self-injury by identifying negative attitudes as a major barrier to seeking help for self-injury. Adding to the work by Rotolone and Martin [21], we were able to distinguish current self-injurers from previous self-injurers and those with no history of self-injury by their attitudes toward seeking help and their intentions to seek help. These factors have not been previously examined.

Overall, and as expected, greater autonomy and more negative attitudes toward help-seeking were associated with lower intentions to seek help from anyone, and attitudes toward help-seeking fully mediated the relationship between autonomy and intentions to seek help for self-injury. As expected current self-injurers were more likely to report more negative attitudes toward seeking help and had lower intentions to seek help than previous self-injurers or non-self-injurers. Previous self-injurers and non-self-injurers were indistinguishable from one another regarding attitudes and intentions to seek help. These findings suggest attitudes and intentions to seek help are factors that may be associated with the cessation of self-injury.

The findings from this study extend our knowledge of what differentiates people who are, and are not, willing to seek help for self-injury. Future research may wish to explore a sense of autonomy, attitudes, and intentions to seek help, in a longitudinal study of self-injury. This would rule out any potential confounds of the current research and better determine specifically why those who have stopped self-injuring have more positive attitudes toward seeking help and are more willing to seek help.

### Implications

We believe the current research has wide implications for clinical practice and community prevention, while also theoretically advancing understanding of the current state of help-seeking for self-injury. Preliminary evidence suggests attitudes toward help-seeking are a key barrier preventing self-injurers from seeking help. As the majority of those who self-injure do not receive help, attitudinal barriers need to be addressed to encourage help-seeking.

## References

[1] Rowe SL, French RS, Henderson C, Ougrin D, Slade M, Moran P (2014). Help-seeking behaviour and adolescent self-harm: a systematic review. Aust N Z J Psychiatry.

[2] Klineberg E, Kelly MJ, Stansfield SA, Bhui KS (2013). How do adolescents talk about selfharm: a qualitative study of disclosure in an ethnically diverse urban population in England. BMC Public Health.

[3] Fadum EA, Stanley B, Rossow I, Mork E, Tormoen AJ, Mehlum L (2013). Use of health services following self-harm in urban versus suburban and rural areas: a national cross-sectional study. BMJ Open.

[4] Berger E, Hasking P, Martin G (2013). ‘Listen to them’: adolescents’ views on helping young people who self-injure. J Adolesc.

[5] Fortune S, Sinclair J, Hawton K (2008). Adolescents’ views on preventing self-harm. a large community study. Soc Psychiatry Psychiatr Epidemiol.

[6] Watanabe N, Nishida A, Shimodera S, Inoue K, Oshima N, Sasaki T (2012). Help-seeking behavior among Japanese school students who self-harm: results from a self-report survey of 18,104 adolescents. Neuropsychiatr Dis Treat.

[7] Fortune S, Sinclair J, Hawton K (2008). Help-seeking before and after episodes of self-harm: a descriptive study in school pupils in England. BMC Public Health.

[8] Ystgaard M, Arensman E, Hawton K, Madge N, Van Heeringen K (2009). Deliberate self-harm in adolescents: comparison between those who receive help following self-harm and those who do not. J Adolesc.

[9] Michelmore L, Hindley P (2012). Help-seeking for suicidal thoughts and self-harm in young people: a systematic review. Suicide and Life-threatening Behavior.

[10] Leavey G, Rothi D, Paul R (2011). Trust, autonomy and relationships: the help-seeking preferences of young people in secondary level schools in London (UK). J Adolesc.

[11] Nada-Raja S, Morrison D, Skegg K (2003). A population-based study of help-seeking for self-harm in young adults. Aust N Z J Psychiatry.

[12] Vogel DL, Wester SR, Larson LM (2007). Avoidance of counselling: psychological factors that inhibit seeking help. Journal of Counseling & Development.

[13] Wrigley S, Jackson H, Judd F, Komiti A (2005). Role of stigma and attitudes toward help-seeking from a general practitioner for mental health problems in a rural town. Aust N Z J Psychiatry.

[14] Sibicky M, Dovidio JF (1986). Stigma of psychological therapy: stereotypes, interpersonal reactions, and the self-fulfilling prophecy. Journal of Counselling Psychology.

[15] Komiti A, Judd F, Jackson H (2006). The influence of stigma and attitudes on seeking help from a GP for mental health problems. Soc Psychiatry Psychiatr Epidemiol.

[16] Long M, Manktelow R, Tracey A (2013). We are all in this together: working towards a holistic understanding of self-harm. J Psychiatr Ment Health Nurs.

[17] Pisani AR, Schmeelk-Cone K, Gunzler D, Petrova M, Goldston DB, Tu X (2012). Associations between suicidal high school students’ help-seeking and their attitudes and perceptions of social environment. Journal of Youth & Adolescence.

[18] Rickwood DJ, Deane FP, Wilson CJ (2007). When and how do young people seek professional help for mental health problems?. Med J Aust.

[19] Wilson C, Rickwood DJ, Bushnell JA, Caputi P, Thomas SJ (2011). The effects of need for autonomy and preference for seeking help from informal sources on emerging adults’ intentions to access mental health services for common mental disorders and suicidal thoughts. Advances in Mental Health.

[20] Wilson C, Deane FP (2012). Brief report: Need for autonomy and other perceived barriers relating to adolescents’ intentions to seek professional mental health care. J Adolesc.

[21] Rotolone C, Martin G (2012). Giving up self-injury: a comparison of everyday social and personal resources in past versus current self-injurers. Archives of Suicide Research.

[22] Fischer EH, Turner JL (1970). Orientations to seeking professional help: development and research utility of an attitude scale. J Consult Clin Psychol.

[23] Wilson C, Deane FP, Ciarrochi J, Rickwood D (2005). Measuring help-seeking intentions: properties of the general-help-seeking questionnaire. Can J Couns.

[24] Goldberg DP, Hillier VF (1979). A scaled version of the general health questionnaire. Psychol Med.

[25] Hayes AF, Preacher KJ (2013). Statistical mediation analysis with a multicategorical independent variable. Br J Math Stat Psychol.

[26] Preacher KJ, Hayes AF (2008). Asymptotic and resampling strategies for assessing and comparing indirect effects in multiple mediator models. Behaviour Research Methods.

[27] Baron RM, Kenny DA (1986). The moderator–mediator variable distinction in social psychological research: conceptual, strategic, and statistical considerations. J Pers Soc Psychol.

[28] Fritz MS, MacKinnon DP (2007). Required Sample Size to Detect the Mediated Effect Psychological Science.

[29] Martin G, Swannell S, Hazell P, Harrison J, Taylor A (2010). Self-injury in Australia: a community survey. Med J Aust.

[30] Muehlenkamp JJ, Gutierrez PM (2004). An investigation of differences between self-injurious behavior and suicide attempts in a sample of adolescents. Suicide and Life-Threatening Behavior.

[31] Hawton K, Rodham K, Evans E, Harriss L (2009). Adolescents who self harm: a comparison of those who go to hospital and those who do not. Child Adolesc Mental Health.

[32] Evans E, Hawton K, Rodham K (2005). In what ways are adolescents who engage in self-harm or experience thoughts of self-harm different in terms of help-seeking, communication and coping strategies?. J Adolesc.

[33] Muehlenkamp J, Walsh B, McDade M (2010). Preventing non-suicidal self-injury in adolescents: the signs of self-injury program. Journal of Youth and Adolescence.

